# Factors contributing to household wealth inequality in under-five deaths in low- and middle-income countries: decomposition analysis

**DOI:** 10.1186/s12889-022-12988-2

**Published:** 2022-04-15

**Authors:** Adeniyi Francis Fagbamigbe, Folashayo Ikenna Peter Adeniji, Oyewale Mayowa Morakinyo

**Affiliations:** 1 Department of Epidemiology and Medical Statistics, Faculty of Public Health, College of Medicine, University of Ibadan, Ibadan, Nigeria; 2 Department of Health Policy and Management, Faculty of Public Health, College of Medicine, University of Ibadan, Ibadan, Nigeria; 3 Department of Environmental Health Sciences, Faculty of Public Health, College of Medicine, University of Ibadan, Ibadan, Nigeria

**Keywords:** Under-5 death, Wealth inequality, Poverty, Low- and middle-income countries, WHO HEAT plus, Fairlie decomposition

## Abstract

**Background:**

The burden of under-5 deaths is disproportionately high among poor households relative to economically viable ones in developing countries. Despite this, the factors driving this inequality has not been well explored. This study decomposed the contributions of the factors associated with wealth inequalities in under-5 deaths in low- and middle-income countries (LMICs).

**Methods:**

We analysed data of 856,987 children from 66,495 neighbourhoods across 59 LMICs spanning recent Demographic and Health Surveys (2010-2018). Under-5 mortality was described as deaths among live births within 0 to 59 months of birth and it was treated as a dichotomous variable (dead or alive). The prevalence of under-five deaths was stratified using household wealth status. A Fairlie decomposition analysis was utilized to investigate the relative contribution of the factors associated with household wealth inequality in under-5 deaths at *p*<0.05. The WHO health equity assessment toolkit Plus was used to assess the differences (D) ratios (R), population attributable risk (PAR), and population attributable fraction (PAF) in household wealth inequalities across the countries.

**Results:**

The proportion of children from poor households was 45%. The prevalence of under-5 deaths in all samples was 51 per 1000 children, with 60 per 1000 and 44 per 1000 among children from poor and non-poor households (*p*<0.001). The prevalence of under-5 deaths was higher among children from poor households than those from non-poor households in all countries except in Ethiopia, Tanzania, Zambia, Lesotho, Gambia and Sierra Leone, and in the Maldives. Thirty-four of the 59 countries showed significantly higher under-5 deaths in poor households than in non-poor households (pro-non-poor inequality) and no significant pro-poor inequality. Rural-urban contexts, maternal education, neighborhood socioeconomic status, sex of the child, toilet kinds, birth weight and preceding birth intervals, and sources of drinking water are the most significant drivers of pro-poor inequities in under-5 deaths in these countries.

**Conclusions:**

Individual-level and neighbourhood-level factors were associated with a high prevalence of under-5 deaths among poor households in LMICs. Interventions in countries should focus on reducing the gap between the poor and the rich as well as improve the education and livelihood of disadvantaged people.

## Background

The reduction of under-five deaths (U5Ds) represents one of the focuses of global health efforts. As articulated in the Sustainable Development Goals (SDGs), countries aim at curtailing U5Ds to at least 25 per 1000 live births by the year 2030 [1]. In line with this goal, considerable progress has been made globally [1–3]. Estimates show that between 1990 and 2019, global U5Ds declined from 93 deaths/1000 live births to 38 deaths/1000 live births, representing about 59 percent decrease during that period [4].

Despite this marked decline, there remains a high burden of U5Ds in many countries of the world. According to the World Health Organization (WHO), about 5.2 million U5Ds occurred in 2019 alone, with the majority of these deaths occurring in low- and middle-income countries (LMICs) [4]. Specifically, the probability of a child dying before age five is 14 times higher in LMICs compared with developed countries, thus, suggesting an enormous gap in the prevalence of U5M [4]. This scenario reflects the conclusion made by Princhett and Summers that the economic prosperity of countries is oftentimes strongly related to their population health outcomes [5]. With a disproportionate burden of U5Ds in developing countries, there is more work to be done to further improve child health outcomes in those countries.

Moreover, evidence in the literature has shown that household wealth is a significant determinant of the risk of U5Ds. A study conducted to estimate the U5Ds by household economic status in LMICs revealed that the probability of U5D in the poorest households is twice that of richer ones [6]. Another study investigated the determinants of U5Ds and revealed that factors like household asset index, maternal literacy level and region had a significant impact on the rate of child mortality [7]. Similarly, van Malderen *et al*. [8] evaluated the socio-economic factors associated with U5Ds in sub-Saharan African countries and found that household economic status, place of residence and the educational level of mother contributed significantly to the burden of U5Ds [8]. The authors noted that the economic background of households was the major contributor to child mortality in some countries. A systematic review of the relationship between income and U5Ds in developing countries reported that after controlling for important covariates, every 10 percent increase in income triggered about 2.8 percent reduction in U5Ds [9]. In general, many other studies have systematically highlighted the impact of the inequality related to family economic status on the odds of mortality in the first 1-5 years of children in LMICs [8, 10–13].

Findings in the reviewed studies suggest that there is a consensus regarding the importance of household economic background in determining child health outcomes. Similarly, the factors associated with inequality in U5Ds have been identified and reported in earlier studies [6–8]. However, the relative contribution of these factors remains unclear in the existing literature, especially in developing countries where the burden of U5Ds is the highest [14]. Therefore, this study aims to decompose factors explaining household wealth inequality in U5Ds in LMICs. This will provide a better understanding that will inform the development of necessary interventions targeted at economically less viable households to reduce child deaths in LMICs. Unlike earlier studies, this study utilized a robust decomposition technique to isolate the relative contributions of different individual-level and neighbourhood-level factors in connection with household wealth inequality in under-5 deaths in LMICs.

## Materials and methods

### Study design and data

The routinely collected Demographic and Health Surveys (DHS) data was used for this analysis. Every five years, the DHS is conducted in each of the participating LMICs. The ICF Macro, the USA in conjunction with the designated organizations such as Statistics boards, Universities, Ministry of Health, etc. in the participating countries collect the data. The surveys nationally representative, population-based and cross-sectional. We merged the DHS conducted between 2010 and 2018 and was released on September 10th, 2020. This study comprised a total of 59 LMICs that matched the inclusion criteria. Data from 856,987 U5M from 66,495 neighborhoods in 59 LMICs was used in the analysis.

### Sampling strategies

In each of the countries where the surveys were conducted, similar clustered multi-stage sampling methodologies were utilised. The sampling frames were mostly based on each country's most recent census figures. The number of levels and their stratification are determined by each country's current geographical and administrative structures. In some of these countries, the multi-stage sampling used regions/states/divisions as the first stage, districts as the second level, and clusters as the final stage. Clusters are defined in the same way in all countries and are also referred to as primary sample units (PSU) [15, 16]. At the last stage, the households were selected from the PSUs.

As a result of uneven population sizes among states/regions/districts within a country, DHS generated and provided sampling weights with the data for each country. The sampling weights were calculated using a multi-stage sampling technique to ensure that the estimates from the samples were representative of the general population. Each country employed a similar set of standardized questionnaires, research protocols, interviewer training procedures, supervision, and execution. The entire specifics of the sample procedures, as well as other data has been reported elsewhere [17].

### Dependent variable

The dependent variable is U5D, which is defined as deaths in the first five years of life among live births [18–20]. It is defined as death occurring between the ages of 0 and 59 months after birth [21]. To ensure the study's accuracy, correctness, and completeness, women were questioned if they had given birth to a child within the previous five years. Those who said yes were then asked to recall the dates of their children's births, followed by a question about whether each of those children was living or deceased at the time of the interview. U5D was calculated using the dates of death or ages at death of the deceased children. As a result, U5D was a binary variable: Dead or Alive before 5th birthday.

#### Household wealth status

The burden of U5Ds was stratified using household wealth status. The DHS survey did not capture participants’ expenditures and incomes but captured an array of assets owned by participants’ households. These assets were used to compute household wealth status within each country by the DHS and released with the data. The quintiles of household wealth were determined using a composite score of assets possessed by households [22]. Detailed report that can be found at dhsprogram.com has further information on the techniques and country-specific assets used to calculate the wealth quintiles in each of the countries. The DHS recommended that the household wealth status derived from asset ownership be used as a proxy for participants' wealth, which may subsequently be interpreted as a measure of their economic or poverty status. The calculated household wealth scores were divided into 20 percent household wealth quintiles. In this study, we re-categorized household wealth quintile into two categories: the first two 20% as poor and the upper three 20% as non-poor. This was to enable the comparison of U5Ds among children from poor and non-poor households. A similar categorization has been used in the literature [23–26].

#### Control variables

Individual-level and neighborhood-level factors were employed as independent variables in the study. These factors have been linked to poverty and childhood mortality in the literature. [6, 8–10, 27].

##### ***Individual-level factors***

Children's characteristics, mothers' profiles, and household variables are the individual-level factors. Weight at birth (very small/small/average), sex (female/male), birth order (1/2/3/4+), birth interval (firstborn/36 months/>=36 months), and if a child is a twin (single/multiple (2+) are all factors considered. Educational level of mother, age of mother, marital status, maternal and paternal employment status, and health insurance status, are among the characteristics of mothers.

The characteristics of households include household's head gender (female/male), access to the media (captured using ownership or access to television, newspaper or radio, at least of these), drinking water sources (either improved/unimproved), toilet type (either improved/unimproved), cooking fuel (clean fuel/biomass), and housing materials (either improved/unimproved) [28–30], and, locations where mothers live (rural or urban). The categorizations of drinking water sources, housing materials, toilet type and cooking fuel as improved or not have been reported in previous studies [18–20, 28–35].

##### Neighbourhood-level factors

The “neighbourhood” is the clustering of children as used in the sampling frames for the surveys. The DHS referred to “cluster” as a common geographical area that contains people that share similar contextual factors [15, 16, 18]. Children in the same cluster were referred to as "neighbours." As a community-level variable, we looked at neighbourhood socioeconomic status (SES). It was a composite variable made up of community education, access to the media, and unemployment rates calculated using the principal component factor approach.

### Statistical analyses

**Fig. 1:**
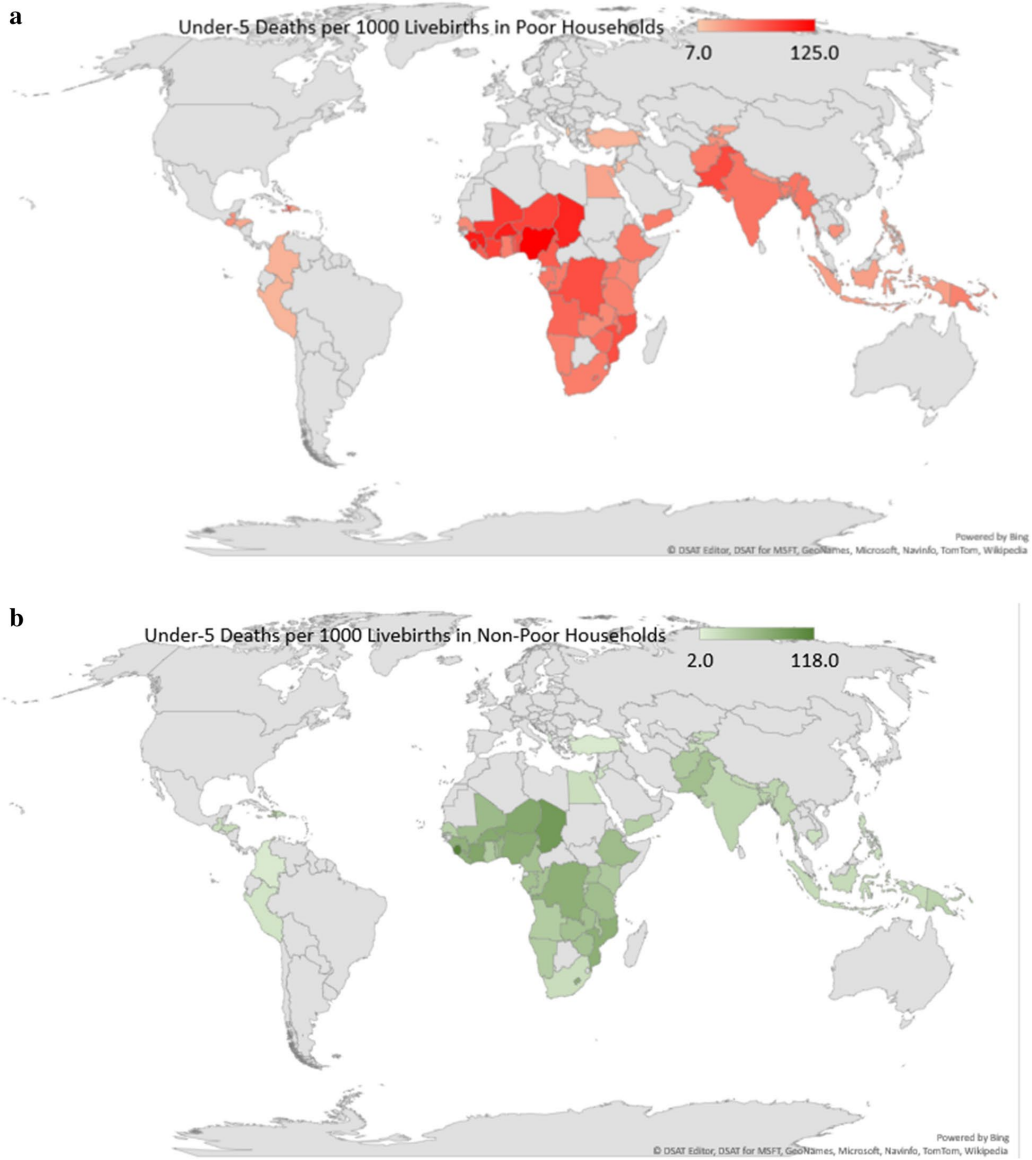
Spatial distribution of under-five deaths among children in poor and non-poor households in the LMIC studied (Source: Authors Drawings)

In this study, descriptive and inferential statistics were used. The country, regions, U5Ds, and other significant features of the children by U5D was depicted using basic descriptive statistics such as maps, graphs, tables, and proportions. Table 1 shows the results of tests of equality in proportions of U5Ds among children from poor and non-poor homes in each country and region. The distribution of the background characteristics of the children by the prevalence of U5Ds among children from poor and non-poor households was reported in Table 2. The spatial distribution of under-five deaths per 1000 live births among children in poor and non-poor households are shown in Fig. 1(a) and (b) respectively. The maps were built in Microsoft Projects 2020. Also, to further examine household wealth inequality in U5Ds, absolute and relative measures of inequality recommended in the WHO Health Equity Assessment Toolkit Plus (HEAT Plus) were utilised [36]. These measures include Difference (D), Ratio (R), Population Attributable Fraction (PAF) and Population Attributable Risk (PAR). The R and D show the relative ratio and absolute difference between two categories within a dimension of inequality (highest and lowest wealth quintile). For D, a positive value indicates that there is pro-non-poor U5Ds and vice versa. The R statistic shows the relative inequality between poor and non-poor households. For an adverse indicator as U5Ds, *R* values equal to 1 indicate that there exists no inequality and values greater than one represent a pro-non-poor U5Ds. The higher this value is, the larger the gap between the poor and non-poor. The PAR is the difference between the most-advantaged subgroup (lowest wealth quintile) and the national average, while PAF is computed by ascertaining the ratio of the national average (μ) and the PAR, multiplied by 100, i.e. PAF = [PAR / μ] * 100. Unlike the R measure of inequality, the PAR and PAF take only negative values for adverse outcomes with higher values reflecting a wider gap between population subgroups. Comprehensive details regarding the computation of these measures have been reported [25]. The R, D. PAF and PAR estimate from household wealth inequality in U5Ds across LMIC using the WHO HEAT Plus are reported in Table 3. The graphical illustrations of the estimates are provided in Fig. 3.

**Table 1:** Distribution of sample characteristics by countries, regions and prevalence of under-five deaths by household wealth inequality in LMIC, 2010–2018

Country	Sample	Year	Number of Communities	Poverty rate	U5Ds per 1000 livebirths		
					Overall	Poor U5D	Non-poor U5D
Overall	856,987		66495	45.1	51	*60	44
Eastern Africa	109,945		6298	45.2	52	*55	50
Burundi	13,192	2011	554	44.0	59	*72	48
Comoros	3,149	2012	252	45.9	42	46	39
Ethiopia	10,641	2016	643	46.8	55	53	57
Kenya	20,964	2014	1593	44.2	44	44	44
Malawi	17,286	2016	850	47.2	49	*52	45
Mozambique	11,102	2011	610	45.0	74	79	71
Rwanda	7,856	2014	492	45.9	39	*46	32
Tanzania	10,233	2015	608	45.4	53	50	55
Uganda	15,522	2016	696	43.5	51	54	49
Middle Africa	76,790		2932	44.3	70	*77	65
Angola	14,322	2016	625	45.9	51	*65	40
Cameroon	9,733	2018	429	45.2	62	*71	53
Chad	18,623	2015	624	42.3	98	*104	93
Congo	9,329	2012	384	46.4	51	53	50
Congo DR	18,716	2014	536	44.1	75	*79	71
Gabon	6,067	2012	334	43.9	53	55	52
Northern Africa	15,848		876	37.6	24	*29	21
Egypt	15,848	2014	876	37.6	24	*29	21
Southern Africa	27,823		2549	44.7	51	53	49
Lesotho	3,138	2014	397	41.4	69	64	73
Namibia	5,046	2013	537	43.7	45	49	41
South Africa	3,548	2016	671	46.2	36	*52	22
Zambia	9,959	2018	545	47.1	49	45	52
Zimbabwe	6,132	2015	399	42.5	57	62	52
Western Africa	147,996		6099	43.7	81	*94	70
Benin	13,589	2018	555	42.0	70	*81	62
Burkina Faso	15,044	2010	573	42.6	89	*108	76
Cote d’Ivoire	7,776	2013	351	47.2	84	88	80
Gambia	8,088	2013	281	42.2	41	38	43
Ghana	5,884	2014	427	43.2	46	51	43
Guinea	7,951	2018	401	45.9	87	*108	68
Liberia	7,606	2013	322	46.6	70	70	70
Mali	9,940	2018	345	42.5	72	*91	58
Niger	12,558	2012	476	40.2	81	85	78
Nigeria	33,924	2018	1389	44.9	97	*125	74
Senegal	6,719	2018	214	45.4	40	44	37
Sierra Leone	11,938	2013	435	44.9	113	109	118
Togo	6,979	2013	330	41.7	63	*75	54
Central Asia	10,558		682	39.2	28	*36	23
Kyrgyz Rep	4,363	2012	316	39.0	26	30	24
Tajikistan	6,195	2017	366	39.3	29	*40	22
South-Eastern Asia	17,716		1851	47.8	26	*33	19
Cambodia	7,165	2014	609	44.4	29	*41	19
Philippines	10,551	2017	1242	50.3	24	*29	19
Southern Asia	338,925		33064	45.8	44	*56	34
Afghanistan	32,712	2015	956	39.7	47	*52	44
Bangladesh	7,886	2014	600	41.9	41	*50	34
India	259,627	2016	28332	47.3	44	*57	32
Indonesia	17,848	2017	1967	40.8	27	*32	24
Maldives	3,106	2016	265	41.9	18	*15	20
Nepal	5,038	2016	383	42.6	34	40	30
Pakistan	12,708	2018	561	42.7	66	*82	54
Western Asia	28,475		2050	46.2	33	*37	29
Armenia	1,724	2016	306	39.6	05	*10	02
Jordan	10,658	2017	964	50.7	17	18	17
Yemen	16,093	2013	780	44.2	45	*52	39
Central America	23,328		1996	47.3	28	*35	22
Guatemala	12,440	2014	856	49.2	31	*41	21
Honduras	10,888	2011	1140	45.0	25	26	23
South America	21,379		4788	49.5	16	*21	12
Colombia	11,759	2015	3386	51.3	15	*21	09
Peru	9,620	2012	1402	47.3	17	20	15
Southern Europe	6,410		688	44.2	10	*14	06
Albania	2,762	2018	652	44.1	04	*07	02
Turkey	3,648	2013	36	44.2	14	*20	09
Caribbean	22,280		1863	45.6	47	*54	40
Dominican Rep	3,714	2013	518	47.0	29	30	28
Haiti	6,530	2016	450	45.8	69	*76	63
Myanmar	4,815	2015	440	52.3	44	*56	31
Timor Leste	7,221	2016	455	40.8	37	*45	32
Oceania	9,514		759	42.0	40	*49	33
Papua NG	9,514	2016	759	42.0	40	*49	33
Total	856,987		66495	45.1	51	*60	44

^*^significant at 5% test of equality of proportion

**Table 2:** Summary of pooled background characteristics of the studied children and prevalence of under-five deaths by household wealth inequality in LMIC, 2010–2018.

Characteristics	sample	%	Poverty rate	U5Ds per 1000 livebirths		
				Overall	poor	Non-poor
Maternal current age						
15-24	254,644	29.7	46.8	53	61	46
25-34	442,799	51.7	43	47	56	40
35-49	159,544	18.6	48.2	61	69	53
Maternal highest educational						
No education	292,866	34.2	62.9	69	72	65
Primary	218,432	25.5	52.2	54	56	53
Secondary+	345,689	40.3	26.3	35	42	32
Media access						
No	340,783	40.5	67	66	67	62
Yes	500,111	59.5	31	43	52	39
Maternal employment						
Employed	324,757	53.3	44.8	61	69	54
Unemployed	284,531	46.7	43.1	45	50	41
Paternal employment						
Employed	541,347	95.8	43.8	55	64	48
Unemployed	23,796	4.2	51.6	48	51	46
Marital status						
Never married	27,341	3.2	37.2	52	52	++52
Currently married	791,531	92.4	45.2	51	60	43
Formerly	38,110	4.4	47.3	63	64	62
Sex of household head						
Male	718,578	83.8	44.9	52	61	44
Female	138,409	16.2	46.2	51	56	46
Covered by health insurance						
No	671,764	87.4	46.1	55	64	48
Yes	96,784	12.6	37.8	33	41	28
Child is twin						
Single birth	834,700	97.4	45.1	47	56	41
Multiple	22,287	2.6	44.2	198	228	175
Sex of child						
Female	417,314	48.7	45.4	48	56	41
Male	439,673	51.3	44.8	55	64	47
Weight at birth						
Average+	671,296	84.0	43.9	45	54	37
Small	92,369	11.6	48.3	67	72	61
Very small	35,374	4.4	49.9	116	114	118
Birth order						
1	243,300	28.4	38.0	48	62	40
2	205,906	24.0	40.5	41	48	35
3	138,761	16.2	46.7	46	52	41
4+	269,020	31.4	54.8	66	7	61
Birth interval						
1st Birth	243,305	28.5	38	48	62	40
<36 months	333,066	39.0	51.5	64	72	55
36+ months	278,326	32.6	44.0	38	41	36
Drinking water						
Unimproved sources	188,610	22.7	66.3	67	70	60
Improved source	641,485	77.3	39.7	47	56	42
Toilet type						
Unimproved sources	416,964	50.3	66.5	63	65	59
Improved source	412,803	49.7	24.5	40	48	38
Cooking fuel						
Unclean/biomass	620,900	76.6	56.5	60	64	56
Clean fuel	189,870	23.4	13.7	30	29	30
Covered by health insurance						
No	261,118	30.5	15.5	40	46	39
Yes	595,869	69.5	59.2	57	62	49
Housing material						
Unimproved material	500,644	62.7	63.3	61	64	55
Improved material	298,152	37.3	19.5	41	49	39
Community SES Disadvantage						
Least	171,506	20.0	19.5	33	41	31
2	171,291	20.0	23.9	46	49	45
3	171,783	20.0	50.7	56	59	53
4	171,392	20.0	58.7	62	67	54
Highest	171,015	20.0	75.6	62	65	53
Total	856,987	100.0	45.1	51	60	*44

++insignificant at 5% test of equality of proportion

**Table 3:** Household wealth inequality in U5Ds in LMIC, 2010–2018 using WHO HEAT Plus

Country	iso3	Year	D (95% CI)	R (95% CI)	PAR (95% CI)	PAF (95% CI)
Afghanistan	AFG	2015	2.4(1.6-3.2)	1.8(1.5-2.2)	-37.1(-47--27.2)	-37.1(-47--27.2)
Albania	ALB	2018	1.3(0.6-2)	0(0-0)	-100(0-0)	-100(0-0)
Angola	AGO	2016	3.7(2.6-4.8)	2.6(1.8-3.8)	-57.4(-72.9--42)	-57.4(-72.9--42)
Armenia	ARM	2016	0.6(-0.2-1.4)	0(0-0)	-100(0-0)	-100(0-0)
Bangladesh	BGD	2014	1.7(0.4-3)	1.6(1.1-2.3)	-31(-50.2--11.8)	-31(-50.2--11.8)
Benin	BEN	2018	2.5(1.3-3.7)	1.5(1.2-1.9)	-34.2(-45.3--23.1)	-34.2(-45.3--23.1)
Burkina Faso	BFA	2010	5.4(4-6.8)	2(1.6-2.4)	-39.1(-48.8--29.3)	-39.1(-48.8--29.3)
Burundi	BDI	2011	4.7(3.4-6)	2.2(1.7-2.7)	-30.4(-42.3--18.5)	-30.4(-42.3--18.5)
Cambodia	KHM	2014	3.9(2.8-5)	5.9(3.3-10.3)	-71.3(-86.4--56.2)	-71.3(-86.4--56.2)
Cameroon	CMR	2018	3(1.4-4.6)	1.7(1.3-2.3)	-31.4(-47.8--15.1)	-31.4(-47.8--15.1)
Chad	TCD	2015	0.1(-1.3-1.5)	1(0.9-1.2)	0(-9.7-9.7)	0(-9.7-9.7)
Colombia	COL	2015	1.5(0.8-2.2)	3.5(1.3-9.8)	-64(-106.2--21.9)	-64(-106.2--21.9)
Comoros	COM	2012	-0.4(-2.8-2)	0.9(0.6-1.5)	0(-42-42)	0(-42-42)
Congo	COG	2012	0.9(-0.7-2.5)	1.2(0.8-1.9)	-27.4(-55.7-0.9)	-27.4(-55.7-0.9)
Congo DR	COD	2014	2.1(0.9-3.3)	1.4(1.1-1.7)	-26(-37.6--14.3)	-26(-37.6--14.3)
Cote d’Ivoire	CIV	2013	1.1(-1.1-3.3)	1.1(0.9-1.5)	-1.1(-21.3-19)	-1.1(-21.3-19)
Dominican Rep	DOM	2013	0.8(-0.8-2.4)	1.4(0.7-3)	-35.7(-79.6-8.2)	-35.7(-79.6-8.2)
Egypt	EGY	2014	1.7(1-2.4)	2.2(1.6-3.1)	-40.1(-56.1--24)	-40.1(-56.1--24)
Ethiopia	ETH	2016	0.5(-0.6-1.6)	1.1(0.9-1.4)	-12(-27.5-3.5)	-12(-27.5-3.5)
Gabon	GAB	2012	0.7(-1.3-2.7)	1.2(0.7-1.8)	-15.5(-50.5-19.4)	-15.5(-50.5-19.4)
Gambia	GMB	2013	1.6(0.3-2.9)	1.6(1.1-2.5)	-35.6(-58.7--12.5)	-35.6(-58.7--12.5)
Ghana	GHA	2014	1.1(-0.7-2.9)	1.3(0.8-1.9)	-13.3(-42.6-16.1)	-13.3(-42.6-16.1)
Guatemala	GTM	2014	3.1(2.2-4)	3.1(2-4.7)	-52.1(-71.2--32.9)	-52.1(-71.2--32.9)
Guinea	GIN	2018	6.9(5.2-8.6)	2.9(2.1-4)	-57.6(-69.9--45.2)	-57.6(-69.9--45.2)
Haiti	HTI	2016	2.9(1-4.8)	1.6(1.1-2.3)	-2.2(-3.7--0.7)	-31.6(-53.0--10.2)
Honduras	HND	2011	1.3(0.3-2.3)	1.7(1.1-2.6)	-25.4(-55.6-4.8)	-25.4(-55.6-4.8)
India	IND	2016	4.2(4-4.4)	3(2.8-3.2)	-53.4(-56.6--50.1)	-53.4(-56.6--50.1)
Indonesia	IDN	2017	1.6(0.9-2.3)	1.7(1.3-2.3)	-21.6(-39.2--4.1)	-21.6(-39.2--4.1)
Jordan	JOR	2017	0.4(-0.7-1.5)	1.3(0.6-2.6)	-14.7(-42-12.5)	-14.7(-42-12.5)
Kenya	KEN	2014	0.3(-0.6-1.2)	1.1(0.9-1.3)	-9.3(-24.8-6.1)	-9.3(-24.8-6.1)
Kyrgyz Rep	KGZ	2012	1.5(-0.1-3.1)	1.7(0.9-3.2)	-23.1(-64.4-18.1)	-23.1(-64.4-18.1)
Lesotho	LSO	2014	1.1(-1.5-3.7)	1.2(0.8-1.9)	-24.9(-52.2-2.4)	-24.9(-52.2-2.4)
Liberia	LBR	2013	2.2(0-4.4)	1.5(0.9-2.3)	-31.9(-60.2--3.5)	-31.9(-60.2--3.5)
Malawi	MWI	2016	0.4(-0.6-1.4)	1.1(0.9-1.3)	-3.1(-17.2-11)	-3.1(-17.2-11)
Maldives	MDV	2016	-0.5(-2.9-1.9)	0.7(0.2-2.7)	0(-154.7-154.7)	0(-154.7-154.7)
Mali	MLI	2018	5.9(4.3-7.5)	2.5(1.9-3.3)	-44.9(-57.3--32.5)	-44.9(-57.3--32.5)
Mozambique	MOZ	2011	1.6(0-3.2)	1.2(1-1.5)	-4.4(-16.9-8.1)	-4.4(-16.9-8.1)
Myanmar	MMR	2015	2.7(1-4.4)	2.1(1.2-3.6)	-43.6(-72.2--15.1)	-43.6(-72.2--15.1)
Namibia	NAM	2013	2.6(0.7-4.5)	1.9(1.1-3.1)	-33.3(-61.6--4.9)	-33.3(-61.6--4.9)
Nepal	NPL	2016	1.7(0.2-3.2)	1.8(1-3.3)	-39.5(-72.2--6.7)	-39.5(-72.2--6.7)
Niger	NER	2012	1.7(0.4-3)	1.3(1.1-1.6)	-27.3(-36.6--18)	-27.3(-36.6--18)
Nigeria	NGA	2018	7.4(6.4-8.4)	2.5(2.1-2.8)	-48.1(-54.2--42)	-48.1(-54.2--42)
Pakistan	PAK	2018	3.6(2.2-5)	1.8(1.4-2.2)	-28.4(-41.1--15.8)	-28.4(-41.1--15.8)
Papua NG	PNG	2016	3.2(1.9-4.5)	2.2(1.6-3)	-29.4(-45.9--13)	-29.4(-45.9--13)
Peru	PER	2012	0.6(-0.4-1.6)	1.4(0.8-2.7)	-21.3(-67.1-24.4)	-21.3(-67.1-24.4)
Philippines	PHL	2017	0.9(-0.1-1.9)	1.5(0.9-2.4)	-23.9(-57.8-10)	-23.9(-57.8-10)
Rwanda	RWA	2014	3(1.7-4.3)	2.3(1.6-3.4)	-40.1(-59--21.2)	-40.1(-59--21.2)
Senegal	SEN	2018	2.6(1-4.2)	2.1(1.2-3.9)	-45.8(-77.7--13.8)	-45.8(-77.7--13.8)
Sierra Leone	SLE	2013	-1.4(-3.3-0.5)	0.9(0.8-1)	0(-12.4-12.4)	0(-12.4-12.4)
South Africa	ZAF	2016	4.3(2.4-6.2)	4.9(1.8-13.6)	-70.4(-103.5--37.2)	-70.4(-103.5--37.2)
Tajikistan	TJK	2017	2.1(0.7-3.5)	2(1.3-3)	-22.7(-45.9-0.5)	-22.7(-45.9-0.5)
Tanzania	TZA	2015	-1.5(-2.9--0.1)	0.7(0.6-1)	0(-19.3-19.3)	0(-19.3-19.3)
Timor Leste	TLS	2016	2.6(1.1-4.1)	2(1.3-2.9)	-27.5(-50.4--4.5)	-27.5(-50.4--4.5)
Togo	TGO	2013	4.1(2.5-5.7)	2.2(1.5-3.2)	-47.7(-64.2--31.2)	-47.7(-64.2--31.2)
Turkey	TUR	2013	0.2(-1.1-1.5)	1.1(0.5-2.8)	-5(-77.5-67.5)	-5(-77.5-67.5)
Uganda	UGA	2016	1.7(0.7-2.7)	1.4(1.1-1.8)	-24.6(-38.7--10.4)	-24.6(-38.7--10.4)
Yemen	YEM	2013	0.8(-0.2-1.8)	1.2(0.9-1.6)	-22(-37.6--6.4)	-22(-37.6--6.4)
Zambia	ZMB	2018	0.1(-1.3-1.5)	1(0.8-1.4)	0(-22.8-22.8)	0(-22.8-22.8)
Zimbabwe	ZWE	2015	3.3(1.6-5)	2(1.4-2.9)	-41.8(-58.6--24.9)	-41.8(-58.6--24.9)

**Fig. 2:**
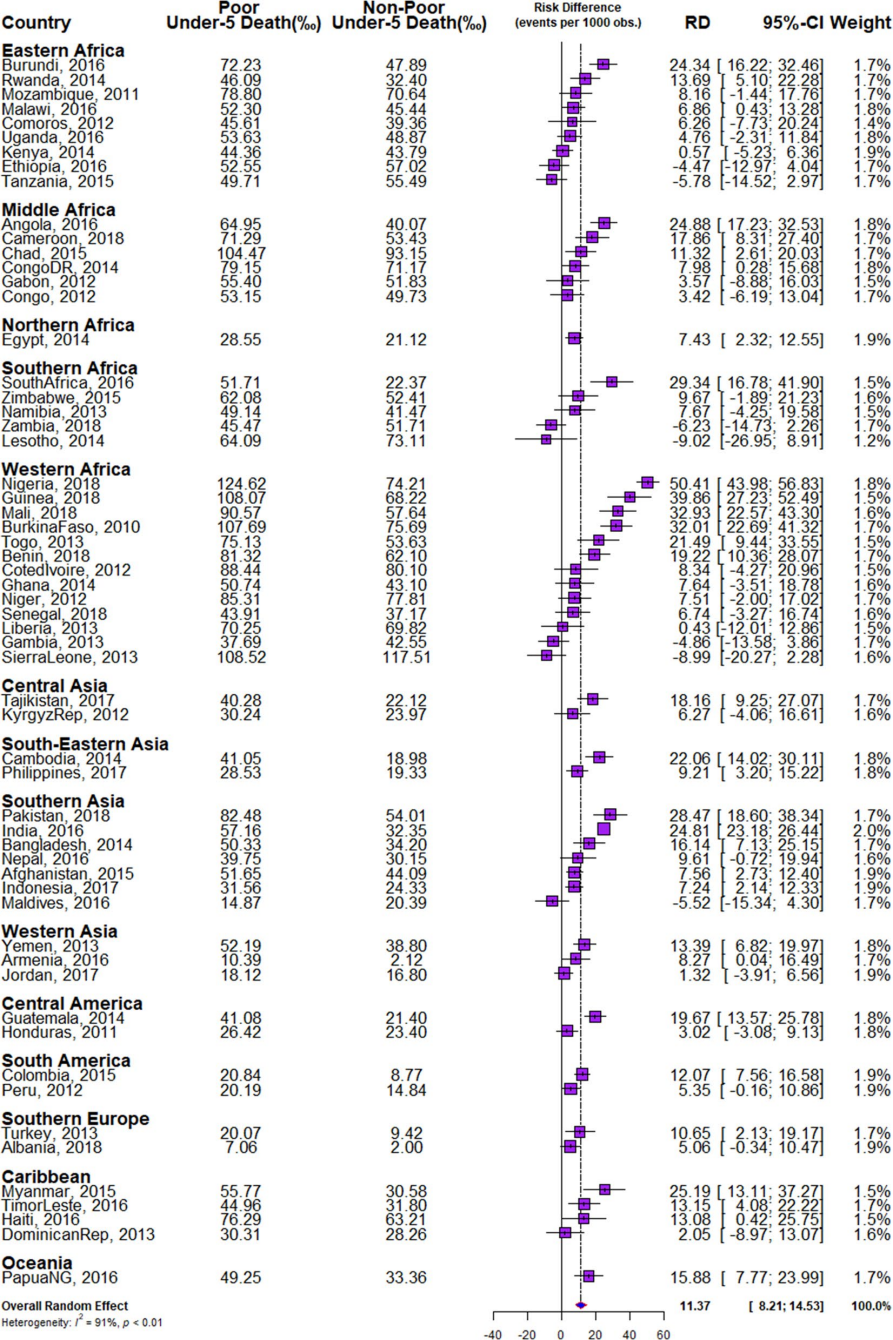
Forest plot of the risk difference in the prevalence of under-five deaths by household wealth inequality in LMIC (Source: Authors Drawings)

**Fig. 3:**
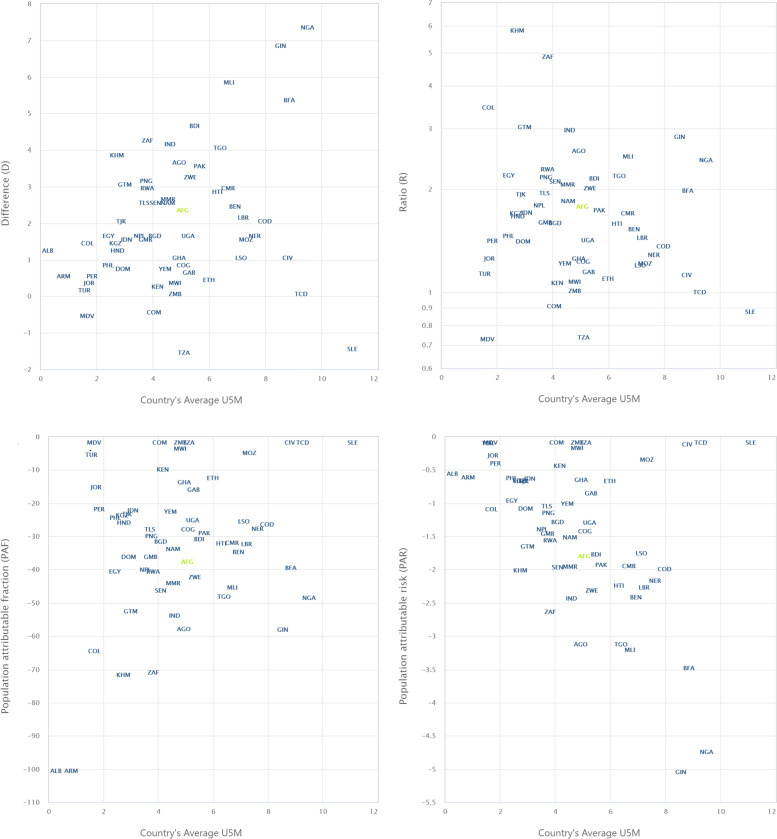
The differences (D) ratios (R), population attributable risk (PAR), and population attributable fraction (PAF) in household wealth inequalities across the LMIC using the WHO HEAT Plus (Abbreviations of the country names are provided in Table 3)

**Fig. 4:**
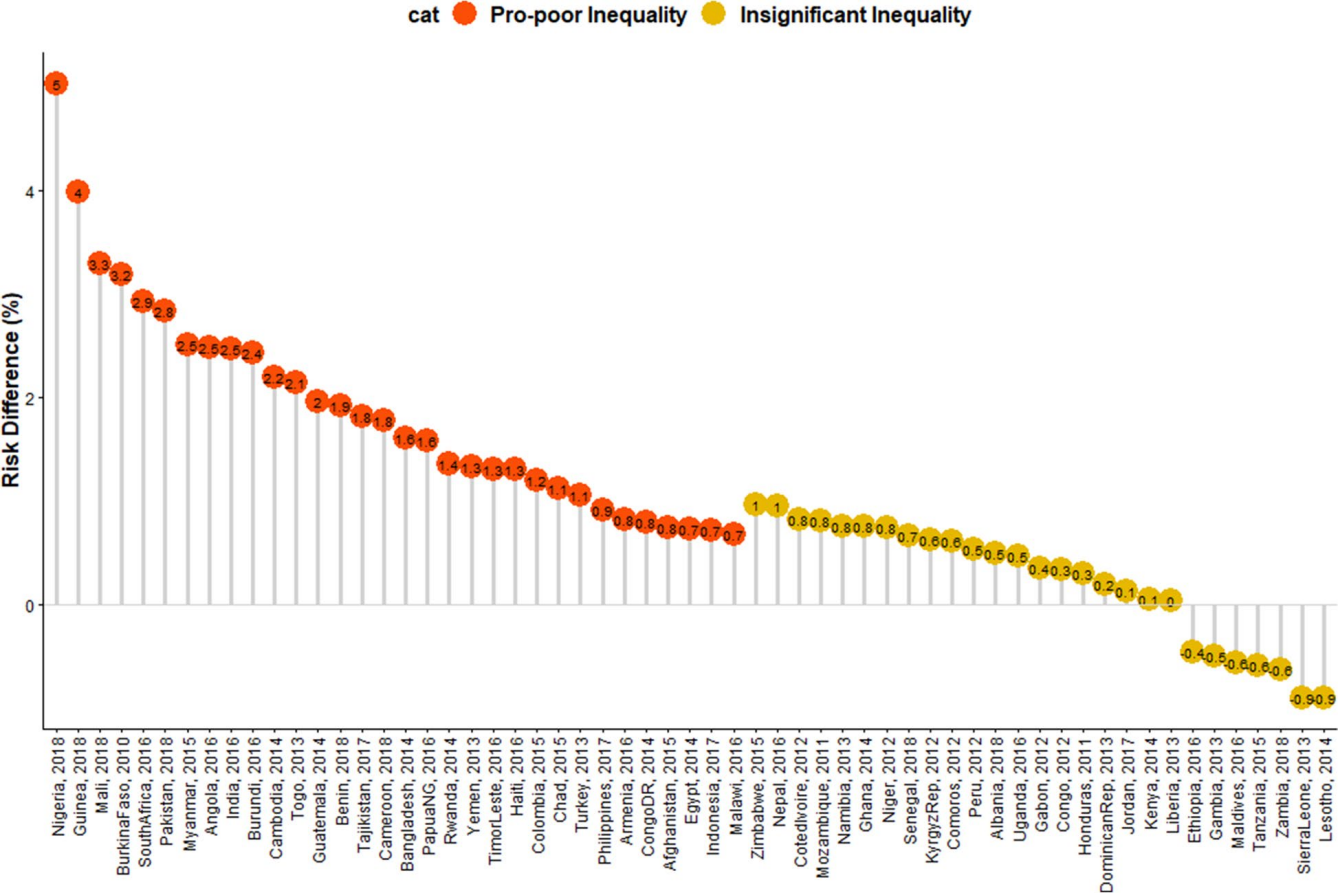
Risk difference in the prevalence of under-five deaths between children from poor and non-poor households in LMICs (Source: Authors Drawings)

**Fig. 5:**
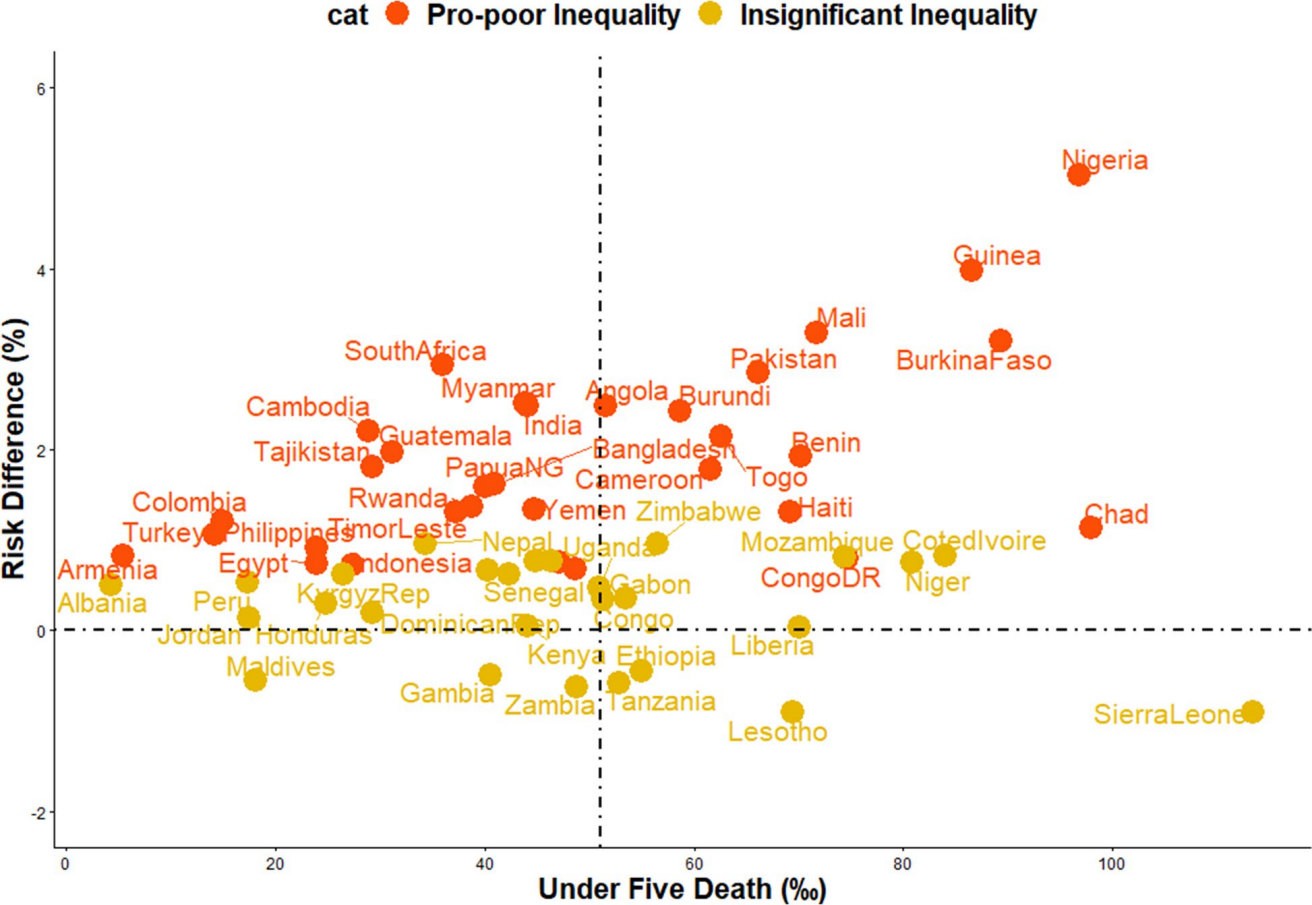
Scatter plot of rate of under-five deaths and risk difference by household wealth inequality in LMICs (Source: Authors Drawings)

We obtained the risk difference (RD) between the risk of U5Ds among children from poor and non-poor households for each country and showed the meta-analysis of these RDs in Fig. 2. We calculated the risk difference in U5D between poor and non-poor households and displayed the results in Fig. 2 as a country-level meta-analysis of U5D prevalence in each of the countries. A random-effects meta-analysis was used based on the assumption that each trial calculates a study-specific actual effect. Using the "metabin" tool in R, the meta-analysis was carried out by identifying the summary measure (SM) as risk difference (RD), the number of fatalities in poor and non-poor households, and the total number of participants for each country, stratified by regions [18]. Scatter and ordered balloon charts were used to show the distributions of the RDs viz-a-viz the prevalence of U5Ds in each country in Figs. 3 and 4. We defined pro-poor inequality as situations in which the RD in U5D is significantly lower among children from poor households than those from non-poor households and pro-non-poor inequality as situations in which the RD in U5D is significantly higher among children children from poor poor households than those from non-poor households [18, 19]. The countries formed 3 groups based on the RDs: countries with pro-poor, insignificant and pro-non-poor inequalities. The “pro-non-poor inequality” and “pro-poor inequality” countries are countries with higher U5D in poor households than in non-poor households and vice versa. Lastly, we fitted adjusted binary logistic regression to the risk of U5Ds among all the pro-poor countries and applied a Fairlie decomposition analysis (FDA) to the inequality in the U5Ds among children from poor and non-poor households and the results were presented in Fig. 5.

We applied sampling weights to all the analyses to control for different cluster sizes and stratifications, as well as to guarantee that our results accurately reflect the target population The "colin" tool in Stata version 16 was used to test for multicollinearity among the independent variables. The variable inflation factor was specified by the command (VIF). The VIF is around 1/(1-R2) and ranges from 1 to Regressing the jth independent variable on other independent variables yields the *R*2-value. All variables with a VIF greater than 2.5 were eliminated from the regression [37]. In several countries, insurance coverage, the employment status of father, access to media, cooking fuel type, and housing material were not reported and were excluded from the decomposition analysis. Prior to performing the decomposition analysis, we conducted a test of heterogeneity of U5D chances across all nations to confirm the presence of heterogeneity. We computed and presented the I-squared and the Mantel-Haenszel (MH) pooled estimate of the odds ratio (OR). We selected the pro-non-poor countries, conducted a homogeneity test among them, and provided the I-squared and MH pooled odds ratio (OR) estimates.

### Decomposition analysis

Several studies on the understanding of factors associated with inequalities in a wide range of health outcomes have adopted the technique of multivariable decomposition analysis [24, 26, 38–40]. Multivariable decomposition analysis is ideal for the quantifications of the contributions of different factors to gaps in an outcome of interest between two groups [41]. It constrains the predicted probability of U5Ds to between 0 and 1. The difference between the predicted probability for one group (say, Group A – poor) using the regression coefficients of the other group (say, Group B – non-poor) and the expected probability for the non-poor group using its regression coefficients is measured in the decomposition analysis [42].

According to Fairlie *et al*., the decomposition of a nonlinear model *Y=F(X)* can be written as:


1


Where ${N}^{A}$ is the sample size for group $J$. Other model details have been reported [18, 19, 31, 33, 43]. The independent contribution of ${X}_{1}$ and ${X}_{2}$ to the gap are expressed as follows:

2 $$\frac{1}{{N}^{B}}X\sum_{i=1}^{{N}^{B}}F\left({\widehat{\alpha }}^{*}+{X}_{1i}^{A}{\widehat{\beta }}_{1}^{*}+{X}_{2i}^{A}{\widehat{\beta }}_{2}^{*}\right)-F\left({\widehat{\alpha }}^{*}+{X}_{1i}^{B}{\widehat{\beta }}_{1}^{*}+{X}_{2i}^{A}{\widehat{\beta }}_{2}^{*}\right).$$

and

3 $$\frac{1}{{N}^{B}}X\sum_{i=1}^{{N}^{B}}F\left({\widehat{\alpha }}^{*}+{X}_{1i}^{B}{\widehat{\beta }}_{1}^{*}+{X}_{2i}^{A}{\widehat{\beta }}_{2}^{*}\right)-F\left({\widehat{\alpha }}^{*}+{X}_{1i}^{B}{\widehat{\beta }}_{1}^{*}+{X}_{2i}^{B}{\widehat{\beta }}_{2}^{*}\right).$$

respectively. Further numerical details have been documented in the literature [42, 44–47]. In this study, the FDA was implemented in STATA version 16 (StataCorp, College Station, Texas, United States of America) using the “Fairlie” command. However, Fairlie’s sequential decomposition has issues with path dependence [42, 44–47], whereby different ordering of variables in the decomposition analysis produces different results. To address this, we checked the robustness of the sensitivity analysis of variable re-ordering randomization. First, we conducted and assessed the performance of 10 different ordering of the variables and tested the sensitivity of decomposition estimates. Secondly, we invoked the “random” option with the “Fairlie” Stata command used in conducting the Fairlie decomposition. In this study, the FDA was implemented in STATA version 16 (StataCorp, College Station, Texas, United States of America).

## Results

The overall proportion of children from poor households irrespective of country of residence was 45%. The prevalence of U5Ds in all samples was 51 per 1000 children. There were significantly different rates of U5Ds across countries at *p*<0.001, with 60 per 1000 and 44 per 1000 among children from poor and non-poor households respectively (Table 1 and Fig. 2). The prevalence of U5Ds among children from poor households ranged from 7 per 1000 live births in Albania to 125 in Nigeria while it ranged from 2 in Albania to 118 in Sierra Leone among children from non-poor households.

The spatial distribution of under-five deaths per 1000 livebirths among children in poor and non-poor households are shown in Figs. 1(a) and 1(b) respectively.

Table 2 shows that the prevalence of U5Ds among children from poor and non-poor households was significantly different across all categories of all the explanatory variables considered in this study except among children of never-married mothers. The widest gaps in the prevalence of U5Ds in poor and non-poor households were among children from multiple births and those with very small birth weights.

### Magnitude and differences in poor-non-poor inequality in U5Ds

The RDs, a measure of inequality in the risk of U5Ds among children from poor and non-poor homes across the 59 nations, are shown in Fig. 2. In all countries except Ethiopia, Tanzania, Zambia, Lesotho, Gambia, and Sierra Leone, and the Maldives, the prevalence of U5Ds was higher among children from poor households than among children from non-poor households. The RDs were considerably greater in 32 countries among children from poor families, but not in any country among children from non-poor households. The distribution of the fixed effects of poor-non-poor RD showed the widest gap in Nigeria (50.4 per 1000 children) followed by Guinea (39.9 per 1000 children). The random-effects irrespective of the child's country of residence was 11.4/1000 (95% confidence interval (CI): 8.2 – 14.5). This indicates that there is significant pro-non-poor inequality in U5Ds in LMICs. India (2.0%) contributed the most weight to the random effect, with 1.9% contributions each in Kenya, Egypt, Afghanistan, Indonesia, Jordan, Colombia, Peru, and Albania while the least weight contribution to the random effect was in Lesotho. The heterogeneity level among the RDs was 91.8% (*p*<0.01).

### The WHO HEAT Plus of R, D, PAR and PAF in household inequalities

Household inequality in U5Ds in 59 countries using the measures recommended in the WHO HEAT Plus showed that there exist wide gaps in U5Ds among poor and rich households. The *D* values indicate that there was a pro-non-poor inequality in U5Ds in the majority of the countries, with the highest gap observed in Nigeria (D=7.4, CI: 6.4-8.4), Guinea (D=6.9, CI: 5.2-8.6), Mali (D=5.9, CI: 4.3-7.5) and South Africa (D=4.3, CI:2.4-6.2). There was a pro-poor inequality in U5Ds in only four countries, Comoros (D=-0.4, CI: -2.8-2), Maldives (D=-0.5, CI: -2.9-1.9), Sierra Leone (D=-1.4, CI: -3.3-0.5) and Tanzania (D=-1.5, CI: -2.9--0.1). The *R* values indicated that there were gaps in U5Ds but the largest gaps were seen in Cambodia (*R *= 5.9, CI: 3.3-10.3) and South Africa (*R *= 4.9, CI: 1.8-13.6). Similarly, the *R* values show that the burden of U5Ds was concentrated among richer households in Comoros (*R *= 0.9, CI: 0.6-1.5), Maldives (*R *= 0.7, CI: 0.2-2.7), Sierra Leone (*R*=0.9, CI: 0.8-1) and Tanzania (*R*=0.7, CI: 0.6-1). This pattern was also observed for the PAR and PAF measures of inequality as shown in Table 3. The visualization of the distribution of these measures in the LMIC is shown in Fig. 3.

### Risk difference and prevalence of under-5 deaths and magnitude of poor-non-poor inequality

Figures 4 and 5 shows the distribution of risk difference of U5Ds by the prevalence of U5Ds in each of the countries. In these charts, significant pro-non-poor inequalities are shown in red colour while insignificant inequities are shown in yellow. There was no significant pro-poor inequality in any country. As shown in the RDs, two of the nine nations in Eastern Africa, four of the six countries in Middle Africa, Egypt in Northern Africa, and six of the thirteen countries in West Africa exhibit significant pro-poor U5Ds inequality. There are two countries in each of Southeast Asia and Western Asia, three in the Caribbean, five in Southern Asia, and one in Southern Africa. Papua New Guinea in Oceania, South America, Central Asia, Central America, and Southern Europe all have high pro-non-poor U5Ds inequality, as seen in Figs. 1, 4, and 5.

### Relationship between the burden of under-5 deaths and magnitude of inequality

We categorized the 59 countries into 4 distinct groups based on the prevalence of U5Ds in each country and based on the magnitude of the RDs which reflects the level of inequality: (i) High prevalence of U5Ds and high pro-non-poor inequality countries which were observed in countries like Nigeria, Guinea, Burkina Faso, Mali and Chad (ii) High prevalence of U5Ds and high pro-poor inequality countries such as Ethiopia, Lesotho and Sierra Leone (iii) Low prevalence of U5Ds and high pro-non-poor inequality countries such as Cambodia, South Africa, Guatemala and Myanmar (iv) Low prevalence of U5Ds and high pro-poor inequality countries such as Maldives, Gambia, and Zambia (Fig. 5).

### Decomposition of poverty inequality in the burden of under-5 deaths

The Mantel-Haenszel (MH) pooled estimate of the odds ratio (OR) of having U5Ds while controlling for the country of residence among children was 1.38 (95% CI: 1.35-1.41) and tested H_o_: OR=1; we estimated z = 32.3 and *p* = 0.000 and (ii) Test of heterogeneity, we estimated *X*^*2*^ = 431.8, degree of freedom (d.f.) = 58, and *p* = 0.000, I-squared (variation in odds ratio (OR) attributable to heterogeneity) = 86.6%. Thirty-four of the 59 countries showed a significant pro-non-poor odds ratio, no significant pro-poor inequality while other countries showed no significant inequality. The 34 countries are Afghanistan, Angola, Bangladesh, Benin, Burkina Faso, Burundi, Cambodia, Cameroon, Chad, Colombia, Cote D’Ivoire, Egypt, Ethiopia, Guatemala, Guinea, Haiti, India, Indonesia Malawi, Mali, Myanmar, Nepal, Niger, Nigeria, Pakistan, Papua New Guinea, Philippines, Rwanda, Senegal, South Africa, Tajikistan, Togo, Turkey and Yemen. A MH OR across these countries was 1.51 (95% CI: 1.47 – 1.54), test of homogeneity of odds ratio was significant with I^2^ = 93.1%, *X*^*2*^ = 155.16, d.f = 33, and *p* = 0.000.

**Fig. 6:**
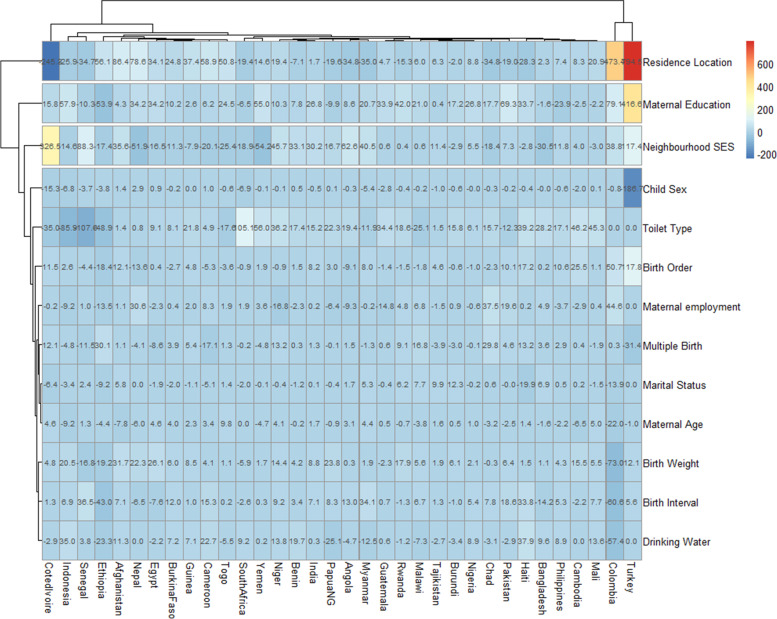
Contributions of differences in the distribution of ‘compositional effect’ of the determinants of under-five deaths to the total gap in household wealth inequality among countries with pro-non-poor inequality in LMIC (Source: Authors Drawings)

Across the 34 countries, the largest contributors to pro-non-poor inequalities in U5Ds among the children are rural-urban differences in the location of residence, maternal education, neighbourhood SES, sex of the child, toilet types, birth weight and preceding birth intervals, sources of drinking water and household wealth. The countries with the largest contributions of these factors are Turkey, Cote D’Ivoire, and Niger. These countries were clustered together while the location of residence, birth order, maternal education, sex of the child, toilet type and maternal employment were clustered together as shown in Fig. 6. The largest contributors to pro-non-poor inequality in Turkey were residence location (473%), birth order (209%) and maternal education (319%). In Cote D’Ivoire, residence location (245%), birth order (213%) and maternal education (35%), contributed the largest to pro-non-poor inequality in U5Ds. Also, the contributions of household wealth to gaps in U5D in poor and non-poor households were shown in Fig. 5 with the highest influence in Turkey, Cote D’Ivoire, Colombia, Ethiopia and Senegal. In general, poor maternal education widens the wealth inequality in child death while better educational attainment closes the gap. Also, living in rural areas, social-economic disadvantaged communities, unimproved toilet type, low birth weight and high birth interval widens wealth inequality in under-five deaths.

## Discussions

The burden of U5Ds is disproportionately higher among the poorest households relative to the richest ones in developing countries. In recent literature, the need for research studies that investigate the relative contributions of the factors associated with inequalities in U5Ds deaths among poor and non-poor households has been well highlighted. This is important to drive the understanding of ways to design policies that will be beneficial for addressing household wealth inequalities in U5Ds which currently exist in LMICs. This study decomposed the individual- and neighbourhood-level factors that have been reported in empirical studies to be associated with wealth inequalities in U5Ds in LMICs [7, 10, 48, 49].

Across all the countries, 45% of the children were from poor households. This is a direct reflection of the economic situation in LMIC where there remains a high burden of child poverty relative to the much lower child poverty rates in wealthier countries [50]. Findings in this study revealed that the average U5Ds rate was 51 per 1000 live births in the 59 LMICs and the mortality rates were significantly different among countries. For poor households, the U5Ds rate was 60 per 1000 live births relative to 44 per 1000 in non-poor households. Also, among poor households, U5Ds was the lowest in Albania (7 per 1000 live births) and the highest in Nigeria (125 per 1000 live births). The disparity in U5Ds in Albania and Nigeria can be hinged on the differences in the level of poverty, population size, education and access to affordable healthcare services. Similarly, for non-poor households, Albania had the lowest U5Ds and the highest in Sierra Leone (118 per 1000 live births). This further reiterates the finding that U5Ds are systematically different among poor and non-poor households [27]. The case of Sierra Leone where there are high U5Ds in both poor and rich households is very worrisome. It followed a different pattern from that observed in other LMICs. This suggests that the economic status of households is less important with regard to the burden of U5Ds in the country. Even though the reduction of income/wealth inequality is a desirable goal of governments globally, other interventions targeted at curtailing U5Ds should be adopted in such countries. In addition, useful lessons can be learnt from countries like Armenia where the prevalence of U5Ds is relatively low just as the gap in U5Ds between children from poor and non-poor households is low as found in this study.

The average estimates across countries revealed in this study represent a slight improvement on the findings published in the work of Chao *et al*. [6] where the average U5Ds was reported to be 64.6 per 1000 live births among poor households and 31.3 per 1000 under-5 children in non-poor families between 1990 and 2016. This is consistent with findings in previous studies that progress has been made in the reduction of U5Ds around the world [51–53]. Despite this noticeable improvement in child health in LMICs, the success needs to be carried forward through the implementation of effective and efficient interventions to match the progress in high-income countries, reduce the relative inequality in U5Ds and guarantee the realization of SDGs by 2030 in developing countries.

Furthermore, the burden of U5Ds was examined by households’ poverty categories across different covariates in all 59 countries. Our findings demonstrated that U5Ds differed significantly across individual and neighborhood-level characteristics, with the biggest disparity seen between poor and non-poor households with multiple births and low birth weights. However, there was no significant difference in the burden of U5Ds among poor and non-poor households by marital status. The increased likelihood of U5Ds due to the small birth weight found in this study has also been reported in earlier research works. A study conducted to examine the mortality risks attributable to preterm and low birth weights in LMICs revealed that children who had small birth weights face a higher risk of mortality compared to those with normal birth weights [54]. Likewise, this finding is supported by the conclusions made in a similar study implemented to investigate the burden and consequences of small birth weight in developing countries. The study showed that having a small birth weight increased the chance of U5Ds in LMICs [55]. The intuition here is that mothers from poorer households may be unable to afford adequate dietary and nutritional intake during pregnancy, among other economic-related deprivations, which can lead to giving birth to children with small birth weights. Consequently, this can generate substantial disparity in the burden of U5Ds among poor and non-poor households.

The inequality in U5Ds observed among rich and poor households was further supported by estimates generated from the measures recommended in the WHO HEAT Plus. In all the measures, D, R, PAF and PAR, the burden of U5Ds was disproportionately higher among poor households, depicting a pro-non-poor inequality in U5Ds.

Moreover, this study utilized a measure of inequality in the probability of U5Ds (the RD of mortality), to assess the risk differences of U5Ds among poor and non-poor households. Under-5 children who live in poor households in 34 LMICs faced higher risks of dying before age 5 compared with their counterparts who are from non-poor households in those countries. Although, this was not the case in some countries- Ethiopia, Tanzania, Zambia, Lesotho, Gambia, Sierra Leone and Maldives. The former scenario is expected since children born in poor families in LMICs are often deprived of basic resources like adequate nutritional intake, access to potable water, access to childhood vaccination/immunization coverage, conducive growing environment (or adequate housing facilities), access to quality healthcare etc. Consequently, the majority of them are predisposed to illnesses emanating from the interaction of those factors which may culminate into U5D. This assertion is corroborated by the conclusions made in the work of Houwelling and Kounst that the economic status of households in LMICs is strongly correlated with the risk of U5D [27]. They further submitted that, as a way pass-through, the relationship between childhood mortality and poverty can be as a result of the impact of economic deprivation on ill-health and can also be the other way round, suggesting a bi-directional relationship [27]. Houwelling *et al* assessed access to skilled maternal care among poor and non-poor households and found that there exist enormous inequalities in the use of professional maternal care services across different income groups in LMICs and that the services provided by nurses and mid-wives appear to favour households in the upper economic stratum relative at the expense of the poor ones [56]. Presumably, this finding may also partly explain the huge gap in child health outcomes among households in the different economic hierarchies. It is important to note that inequalities in U5D as shown in the RDs in U5Ds followed a similar pattern in all the countries apart from Sierra Leone where the burden of U5D is high both among poor and rich households. This suggests that there may be other fundamental issues affecting child health outcomes in the country.

In general, our study showed evidence of significant differences in the risk of U5Ds among children from poor and non-poor households in the 59 LMICs countries and this gap was widest in Nigeria (50.4 per 1000 live births) and Guinea (39.9 per 1000 live births). Overall, the RD across countries was 11.4 per 1000 under-5 children. The differences in the risk of U5Ds across the countries could be as a result of the differences in child poverty in those countries which further emphasise the earlier assertions made regarding the strong link between household economic status and the risk of U5Ds. Another probable justification for the differences in the poor-rich inequalities in U5Ds can be hinged on other country-level factors such as availability of social protection, the extent of income inequalities within countries, healthcare financing infrastructure etc. [27].

More importantly, the decomposition analysis in this study found that factors like rural-urban contexts, maternal education, neighbourhood SES, sex of the child, birth weight, preceding birth intervals, sources of drinking water and household wealth, explain the majority of the inequalities in U5Ds among poor and non-poor households. These factors either contribute to the widening or shrinking of this gap. For instance, this study showed that the location of households (i.e. rural or urban) contributed the highest to household wealth inequalities in U5Ds. A possible reason for this is that households living in urban locations usually have access to good healthcare facilities and better social infrastructure compared with households who reside in rural centres. As a result of this, children born in urban areas will likely have higher survival rates relative to those born in rural centres. Likewise, higher educational attainment of mothers has a positive impact on child health outcomes and thus contributes to the reduction in the gap existing between the poor and the rich with regards to the outcome of U5Ds. Intuitively, educated mothers are more likely to have access to higher income from paid employment and a better standard of living compared with those who are not educated. Another advantage of education among mothers is that it ensures that mothers have better childbearing practices and this will often be reflected in child health outcomes. The implication of this is that governments in developing countries should ensure that the female child has access to quality education as much as their male counterparts as this will lead to better child outcomes in those countries in the longer term.

Moreover, this study indicated that neighbourhood SES also contributed to the gap in U5Ds among poor and rich households. This finding is supported by the evidence revealed in a conducted to examine the pathways of the impacts of neighbourhood SES on the outcome of childbirth [44]. The study showed that neighbourhood SES had a direct effect on the occurrence of preterm birth and low birth weight among children. Another study has also reported that poor birth outcomes are higher among households in the poorest income stratum [45]. Apart from child sex and maternal age, other factors contributing to household wealth inequality in U5Ds like toilet type, birth order, maternal employment, multiple births, birth interval and drinking water are related to the wealth status of households in a way. For the majority of the countries included in this study, this finding further reiterates the need to develop and implement policies that will improve the living standards of households in those countries.

Overall, the contributions of the aforementioned factors to the pro-non-poor disparity in U5Ds varied across countries. For instance, in Cote D’Ivoire, the location of household, birth order and maternal education had the highest contribution while the household wealth, neighbourhood SES, birth interval, birth weight and maternal education contributed the highest to U5Ds in Senegal. This presupposes that interventions to address inequalities in U5Ds among poor and non-poor households in LIMCs should be country-specific in such a way that policies in individual countries are designed with the consideration of the factors contributing the largest to the disparity in U5Ds among poor and rich households. It is important to note that majority of the aforementioned determinants of U5Ds are related to the poverty status of households. For example, poorer households are often more concentrated in rural locations compared with economically more viable families who more often than not reside in urban centres where there is better access to quality healthcare relative to rural centres. This can also be said about other factors that are associated with U5M with regards to their link to poverty, apart from the gender of the child which is typically a biological factor. Therefore, efforts geared towards improving child health in developing countries will have a lot to do with reducing income inequality in the general population. Specifically, interventions targeted at reducing the enormous burden of U5Ds among poor families will need to focus on fostering a system where households, irrespective of their economic status, have access to the factors that improve child health outcomes. As recommended by WHO, factors such as exclusive breastfeeding of infants, access to essential nutrients as well as micronutrients, good awareness and knowledge of the signs that portray danger for under-five children, adequate access to hygiene, portable water and hygiene and immunization, are important for reducing U5Ds and improving child health outcomes, although these are somewhat related to the economic status of households [1].

This study revealed that some countries- Maldives, Armenia, Jordan, Egypt, Philippines, Hondurans, Colombia, Peru and Turkey, have less than 25 U5Ds per 1000 live births targeted in the SDGs. However, other countries have higher U5Ds. In particular, there are some countries with a high prevalence of U5Ds and high-risk differences between children from poor and non-poor households. These countries are Nigeria, Guinea, Burkina Faso, Mali, Chad, Pakistan and Benin. There is a need for these countries to develop and implement policy interventions that draw lessons from the countries (such as Jordan, Albania, Armenia and Peru) where both the risk differences and U5Ds are low. Likewise, countries with high poverty rates and large population sizes will require deliberate efforts geared towards reducing the absolute inequalities between the poor and the rich and in so doing, curtail the social gradient in health as reflected in child health outcomes in those countries. Sierra Leone is an example of countries with a very worrisome situation, insignificant risk difference between children from poor and non-poor households notwithstanding. The level of U5Ds in both types of households in Sierra Leone exceeded 100 per 1000 deaths. Such countries will need greater efforts to reduce U5Ds.

### Study limitations

This study has some limitations which are majorly data issues. The DHS survey was conducted at different times across the 59 LMICs and this may introduce bias in the comparison of U5Ds. Similarly, recall bias may have occurred as mothers were made to recall past events. There is also the possibility of under-reporting of U5Ds in some countries which is often related to cultural taboos forbidding parents from reporting the deaths of their children. Another limitation is that maternal weight or BMI would be useful as the control variables, however, this was not included because maternal height and weight were inadequately reported in the DHS. Only 35% of mothers have the two variables. In addition, FDA is useful for estimating the relative contributions of factors on the outcome of U5Ds. However, the technique does not account for the clustering and stratification elements of the DHS. It is therefore not unlikely that this may have had some impact on the results generated. Nonetheless, FDA is a reliable way for determining the contributions of various factors to gaps in a desired outcome between two groups, and this decomposition technique is an improvement over the Blinder Oaxaca decomposition method.

## Conclusion and recommendation

This study found that there are systematic differences in the burden of U5Ds among households and showed that this burden is disproportionately higher among poor families relative to non-poor ones. Likewise, there is evidence that children born in economically less viable households have higher risks of dying before age 5 compared with those who are from rich households. The decomposition analysis revealed that factors such as rural-urban contexts, maternal education, neighbourhood socioeconomic status, sex of the child, birth weight, preceding birth intervals and sources of drinking water are the main contributors to pro-non-poor inequalities in U5Ds in 34 countries out of the 59 LMICs included in this study. The majority of these factors are related to the economic status of households. As such, there is an increasing need to address this inequality in child health outcomes and a greater gain in this endeavour will be achieved by developing policies that are country-specific and targeted at households in the lower-income strata. Poverty reduction in addition to addressing the factors identified in this study across the countries with significant pro-non-poor inequalities could help reduce U5Ds. Nonetheless, in Sierra Leone, this study revealed that the burden of U5Ds is equally high among poor and rich households. The implication of this is that countries with a similar scenario as the one observed in Sierra Leone where the burden of U5Ds is high among the poor and the rich will have to go beyond the reduction of wealth-related inequalities to adopting other country-specific strategies to reduce U5Ds. In doing this, important lessons can be learnt from the strategies adopted in Maldives, Armenia, Jordan, Egypt, Philippines, Hondurans, Colombia, Peru and Turkey, where the prevalence of U5Ds are relatively low. This study has contributed to the body of knowledge through the identification of factors that contribute to the differences in U5D between poor and rich households.

## Data Availability

The datasets generated and/or analysed during the current study are available in the DHS repository, http://dhsprogram.com with Accession number 140625.

## Abbreviations

CI: Confidence Interval
DHS: Demographic and Health Survey
FDA: Fairlie Decomposition Analysis
IRB: Institutional Review Board
LMIC: Low- And Middle-Income Countries
PSU: Primary Sample Unit
RD: Risk Difference
SES: Socioeconomic Status
U5C: Under-Five Children
U5Ds: Under-five deaths
U5M: Under-five Mortality
UNICEF: United Nations International Children’s Emergency Fund
WHO: World Health Organization

## References

[1] World Health Organization. World health statistics overview 2019: monitoring health for the SDGs, sustainable development goals. World Health Organization; 2019.

[2] Wardlaw T, You D, Hug L, Amouzou A, Newby H (2014). UNICEF Report: Enormous progress in child survival but greater focus on newborns urgently needed. Reprod Health.

[3] Sharrow D, Sun Y, Marcusanu A, You D, Mathers C, Hogan D, Ho J, Mahanani WR, Suzuki E. Levels and trends in child mortality. Estimates developed by the UN Inter-Agency Group for Child Mortality Estimation (IGME) Report by UNICEF, WHO and world bank. 2017.

[4] Who.int. 2021. Under-five mortality rate. [online] Available at https://www.who.int/data/nutrition/nlis/info/under-five-mortality-rate. Accessed 26 Mar 2022.

[5] Pritchett L, Summers LH. Wealthier is healthier. World Bank Publications; 1993.

[6] Chao F, You D, Pedersen J, Hug L, Alkema L (2018). National and regional under-5 mortality rate by economic status for low-income and middle-income countries: a systematic assessment. Lancet Glob Heal..

[7] Mohammad A, Akib Mohammad K, Tabassum T (2016). The Impact of Socio-Economic and Demographic Factors on Under-Five Child Mortality in Bangladesh. Imp J Interdiscip Res IJIR.

[8] Van Malderen C, Amouzou A, Barros AJD, Masquelier B, Van Oyen H, Speybroeck N (2019). Socioeconomic factors contributing to under-five mortality in sub-Saharan Africa: a decomposition analysis. BMC Public Health..

[9] O’Hare B, Makuta I, Chiwaula L, Bar-Zeev N (2013). Income and child mortality in developing countries: A systematic review and meta-analysis. J R Soc Med..

[10] Gebretsadik S, Gabreyohannes E (2016). Determinants of Under-Five Mortality in High Mortality Regions of Ethiopia: An Analysis of the 2011 Ethiopia Demographic and Health Survey Data. Int J Popul Res..

[11] Jain N, Singh A, Pathak P. Infant and child mortality in India: trends in inequalities across economic groups. J Popul Res. 2013;30(4):347–65.

[12] Bendavid E. Changes in child mortality over time across the wealth gradient in less-developed countries. Pediatrics. 2014;134(6):e1551–9. 10.1542/peds.2014-2320 PMC4243072 25384496

[13] Quentin W, Abosede O, Aka J, Akweongo P, Dinard K, Ezeh A (2014). Inequalities in child mortality in ten major African cities. BMC Med.

[14] Seneff Stephanie (2016). Statins and Myoglobin: How Muscle Pain and Weakness Progress to Heart, Lung and Kidney Failure. Br Med Bull..

[15] ICF International. Demographic and Health Survey: Sampling and Household Listing Manual. Calverton; 2012. https://www.dhsprogram.com/pubs/pdf/DHSM4/DHS6_Sampling_Manual_Sept2012_DHSM4.pdf. Accessed 21 Jun 2019.

[16] Croft TN, Marshall AMJ, Allen CK. Guide to DHS Statistics. 2018. https://dhsprogram.com/pubs/pdf/DHSG1/Guide_to_DHS_Statistics_DHS-7.pdf. Accessed 21 Jun 2019.

[17] Dhsprogram.com. 2021. The DHS Program - Quality information to plan, monitor and improve population, health, and nutrition programs. [online] Available at: https://dhsprogram.com/. Accessed 26 Mar 2022.

[18] Fagbamigbe AF, Morakinyo OM, Balogun FM (2021). Sex inequality in under-five deaths and associated factors in low and middle-income countries: a Fairlie decomposition analysis. BMC Public Health..

[19] Morakinyo OM, Fagbamigbe AF, Adebowale AS (2022). Decomposition of factors associated with housing material inequality in under-five deaths in low and middle-income countries. Arch Public Heal..

[20] Fagbamigbe AF, Nnanatu CC. Modelling the Spatial Distribution and the Factors Associated with Under-Five Mortality in Nigeria. Spat Demogr. 2021;1–28. 10.1007/s40980-021-00078-7

[21] Demographic N. Health Survey 2013. National Population Commission (NPC)[Nigeria] and ICF International. Abuja, Nigeria, and Rockville, Maryland, USA: NPC and ICF International.

[22] Vyass S, Kumaranayake L (2006). Constructing Socioeconomic Status Indexes: How to Use Principal Component Analysis. Health Policy Plan..

[23] GBD Diarrhoeal Diseases Collaborators (2017). Estimates of global, regional, and national morbidity, mortality, and aetiologies of diarrhoeal diseases: a systematic analysis for the Global Burden of Disease Study 2015. Lancet Infect Dis..

[24] Ndwandwe D, Uthman OA, Adamu AA, Sambala EZ, Wiyeh AB, Olukade T (2018). Decomposing the gap in missed opportunities for vaccination between poor and non-poor in sub-Saharan Africa: A Multicountry Analyses. Hum Vaccines Immunother..

[25] Novignon J, Aboagye E, Agyemang OS, Aryeetey G (2015). Socioeconomic-related inequalities in child malnutrition: evidence from the Ghana multiple indicator cluster survey. Health Econ Rev..

[26] Almasian-Kia A, Goodarzi S, Asadi H, Khosravi A, Rezapour A. A Decomposition Analysis of Inequality in Malnutrition among Under-Five Children in Iran: Findings from Multiple Indicator Demographic and Health Survey, 2010. Iran J Public Health. 2019;48:748–57. http://www.ncbi.nlm.nih.gov/pubmed/31110986. Accessed 7 Jul 2019. PMC6500533 31110986

[27] Houweling TA, Kunst AE. Socio-economic inequalities in childhood mortality in low-and middle-income countries: a review of the international evidence. British Med Bull. 2010;93(1):7–26. 10.1093/bmb/ldp048 20007188

[28] Morakinyo OM, Fagbamigbe AF (2017). Neonatal, infant and under-five mortalities in Nigeria: An examination of trends and drivers (2003–2013). PLoS One.

[29] Morakinyo OM, Fagbamigbe AF, Adebowale AS (2022). Decomposition of factors associated with housing material inequality in under-five deaths in low and middle-income countries. Arch Public Heal.

[30] Fagbamigbe AF, Oyinlola FF, Morakinyo OM, Adebowale AS, Fagbamigbe OS, Uthman AO (2021). Mind the gap: what explains the rural-nonrural inequality in diarrhoea among under-five children in low and medium-income countries?. A decomposition analysis. BMC Public Health.

[31] Fagbamigbe AF, Ologunwa OP, Afolabi EK, Fagbamigbe OS, Uthman AO (2021). Decomposition analysis of the compositional and contextual factors associated with poor-non-poor inequality in diarrhoea among under-five children in low- and middle-income countries. Public Health..

[32] Fagbamigbe AF, Kandala N-B, Uthman OA (2020). Mind the gap: What explains the poor-non-poor inequalities in severe wasting among under-five children in low- and middle-income countries?. Compositional and structural characteristics. PLoS One..

[33] Fagbamigbe AF, Adebola OG, Dukhi N, Fagbamigbe OS, Uthman OA (2021). Exploring the socio-economic determinants of educational inequalities in diarrhoea among under-five children in low-and middle-income countries: a Fairlie decomposition analysis. Arch Public Heal..

[34] Fagbamigbe AF, Uthman AO, Ibisomi L (2021). Hierarchical disentanglement of contextual from compositional risk factors of diarrhoea among under-five children in low-and middle-income countries. Sci Rep..

[35] Fagbamigbe AF, Oyinlola FF, Morakinyo OM, Adebowale AS, Fagbamigbe OS, Uthman AO (2021). Mind the gap: what explains the rural-nonrural inequality in diarrhoea among under-five children in low and medium-income countries?. A decomposition analysis. BMC Public Health..

[36] Hosseinpoor AR, Schlotheuber A, Nambiar D, Ross Z, Ng N (2018). Global Health Action Health Equity Assessment Toolkit Plus (HEAT Plus): software for exploring and comparing health inequalities using uploaded datasets Health Equity Assessment Toolkit Plus (HEAT Plus): software for exploring and comparing health inequal. Taylor Fr.

[37] Curtis SMK, Ghosh SK (2011). A bayesian approach to multicollinearity and the simultaneous selection and clustering of predictors in linear regression. J Stat Theory Pract..

[38] Asuman D, Ackah CG, Enemark U (2018). Inequalities in child immunization coverage in Ghana: evidence from a decomposition analysis. Health Econ Rev.

[39] Fagbamigbe AF, Kandala NB, Uthman AO. Demystifying the factors associated with rural – urban gaps in severe acute malnutrition among under - five children in low - and middle - income countries : a decomposition analysis. Sci Rep. 2020;10:1–15. 10.1038/s41598-020-67570-w. 10.1038/s41598-020-67570-w PMC7341744 32636405

[40] Fagbamigbe AF, Kandala NB, Uthman OA (2020). Decomposing the educational inequalities in the factors associated with severe acute malnutrition among under-five children in low- and middle-income countries. BMC Public Health..

[41] Powers DA, Yoshioka H, Yun M (2011). mvdcmp: Multivariate Decomposition for nonlinear response models. Stata J..

[42] Fairlie RW (2017). Addressing Path Dependence and Incorporating Sample Weights in the Nonlinear Blinder-Oaxaca Decomposition Technique for Logit.

[43] Fagbamigbe AF, Bello S, Salawu MM, Afolabi RF, Gbadebo BM, Adebowale AS. Trend and decomposition analysis of risk factors of childbirths with no one present in Nigeria, 1990–2018. BMJ Open. 2021;11:e054328. 10.1136/BMJOPEN-2021-054328. 10.1136/bmjopen-2021-054328 PMC8663083 34887282

[44] Norman G, Pedley S, Takkouche B (2010). Effects of sewerage on diarrhoea and enteric infections: a systematic review and meta-analysis. Lancet Infect Dis..

[45] Fairlie RW. An Extension of the Blinder-Oaxaca Decomposition Technique to Logit and Probit Models. Yale; 2003. http://ssrn.com/abstract=497302.

[46] Fairlie RW (1999). The Absence of the African-American Owned Business: An Analysis of the Dynamics of Self-Employment. J Labor Econ..

[47] Jann B. Fairlie: Stata module to generate nonlinear decomposition of binary outcome differentials. 2006;:1. http://ideas.repec.org/c/boc/bocode/s456727.html. Accessed 6 Apr 2020.

[48] Aheto JMK (2019). Predictive model and determinants of under-five child mortality: Evidence from the 2014 Ghana demographic and health survey. BMC Public Health..

[49] Van Malderen C, Van Oyen H, Speybroeck N. Contributing determinants of overall and wealth-related inequality in under-5 mortality in 13 African countries. J Epidemiol Community Health. 2013;67(8):667–76. 10.1136/jech-2012-202195 23704052

[50] Adamson P, Micklewright J, Schnepf S, Wright A. A League Table of Child Maltreatment Deaths in Rich Nations. Innocenti Report Card. Issue No. 5. UNICEF. 3 United Nations Plaza, New York, NY 10017; 2003.

[51] Wang H, Liddell CA, Coates MM, Mooney MD, Levitz CE, Schumacher AE, Apfel H, Iannarone M, Phillips B, Lofgren KT, Sandar L. Global, regional, and national levels of neonatal, infant, and under-5 mortality during 1990–2013: a systematic analysis for the Global Burden of Disease Study 2013. The Lancet. 2014;384(9947):957–79. 10.1016/S0140-6736(14)60497-9 PMC4165626 24797572

[52] Golding N, Burstein R, Longbottom J, Browne AJ, Fullman N, Osgood-Zimmerman A, Earl L, Bhatt S, Cameron E, Casey DC, Dwyer-Lindgren L. Mapping under-5 and neonatal mortality in Africa, 2000–15: a baseline analysis for the Sustainable Development Goals. The Lancet. 2017;390(10108):2171–8. 10.1016/S0140-6736(17)31758-0 PMC5687451 28958464

[53] Mejía-Guevara I, Zuo W, Bendavid E, Li N, Tuljapurkar S (2019). Age distribution, trends, and forecasts ofunder-5 mortality in 31 sub-saharan africancountries: A modeling study. PLoS Med.

[54] Katz J, Lee ACC, Kozuki N, Lawn JE, Cousens S, Blencowe H (2013). Mortality risk in preterm and small-for-gestational-age infants in low-income and middle-income countries: A pooled country analysis. Lancet..

[55] Lee ACC, Kozuki N, Cousens S, Stevens GA, Blencowe H, Silveira MF (2017). Estimates of burden and consequences of infants born small for gestational age in low and middle income countries with INTERGROWTH-21 st standard: Analysis of CHERG datasets. BMJ..

[56] Houweling T, Ronsmans C, Campbell O, Kunst A (2007). Huge poor-rich inequalities in maternity care: an international comparative study of maternity and child care in developing countries. Bull World Health Organ..

